# Expanded description of *Neoechinorhynchus (Hebesoma) manubrianus* (Acanthocephala: Neoechinorhynchidae) from marine fish in Halong Bay, Vietnam

**DOI:** 10.1051/parasite/2012193267

**Published:** 2012-08-15

**Authors:** O.M. Amin, R.A. Heckmann

**Affiliations:** 1 Institute of Parasitic Diseases (IPD), 11445 E. Via Linda # 2-419 Scottsdale, Arizona 85259 USA; 2 Department of Biology, Brigham Young University Provo, Utah 84602 USA

**Keywords:** Acanthocephala, *Neoechinorhynchus manubrianus*, Neoechinorhynchidae, Halong Bay, Vietnam, expanded description, SEM, Acanthocephala, *Neoechinorhynchus manubrianus*, Neoechinorhynchidae, Baie d’Halong, Viêt-Nam, description, MEB

## Abstract

*Neoechinorhynchus manubrianus* Amin, Ha & Ha, 2011 (Acanthocephala: Neoechinorhynchidae) (formerly *Neoechinorhynchus manubriensis* Amin, Ha & Ha, 2011), was recently described based on optical microscopy of four males and two females (none was gravid) from caroun croaker, *Johnius carouna* (Cuvier), flower croaker, *Nibea albiflora* (Richardson), and silver croaker, *Pennabia argentata* (Houttuyen) (Sciaenidae) in Halong Bay, Vietnam. Subsequently, many more specimens became available from *N. albiflora* that were studied using SEM. SEM studies showed many additional features that were not possible to discern with optical microscopy. These included the prominent angulation of the anterior trunk, the presence of (1) anterio-dorsal and (2) undulating mid-lateral fin-like protrusions of the body wall, uniquely shaped eggs as well as details of trunk micropores, proboscis, bursa, and female gonopore. Microscopical examination of eggs from the new collection demonstrated the presence of polar prolongation of fertilization membrane which places *N. manubriensis* in the subgenus *Hebesoma*. The features of trunk angulation, trunk fins, and egg morphology further distinguish *N. manubriensis* from all other species of *Neoechinorhynchus* Stiles and Hassall, 1905 from Vietnam or from any where else in the world.

## Abstract (fr)

*Neoechinorhynchus manubrianus* Amin, Ha et Ha, 2011 (Acanthocephala: Neoechinorhynchidae) (auparavant *Neoechinorhynchus manubriensis* Amin, Ha & Ha, 2011) a été décrit récemment en microscopie optique à partir de quatre males et deux femelles (non gravides) récoltés sur *Johnius carouna* (Cuvier), *Nibea albiflora* (Richardson) et *Pennabia argentata* Houttuyen) (Sciaenidae) de la Baie d’Halong, au Viêt-Nam. Par la suite, de nombreux autres spécimens de *N. albiflora* ont été étudiés en microscopie électronique à balayage (MEB). Ainsi, d’autres caractéristiques qu’il n’était pas possible de discerner en microscopie optique ont été rapportées. L’examen microscopique des oeufs de la nouvelle collection a montré la présence d’une prolongation polaire de la membrane de fertilisation, ce qui permet de situer *N. manubriensis* dans le sous-genre *Hebesoma*. L’angulation antérieure du tronc, les caractéristiques des nageoires et la morphologie des oeufs distinguent plus avant *N. manubriensis* de toute les autres espèces de *Neoechinorhynchus* Stiles et Hassall, 1905, du Viêt-Nam ou de toute autre région du monde.

The recent description of *Neoechinorhynchus manubrianus* Amin, Ha & Ha, 2011 (Acanthocephala: Neoechinorhynchidae) (formerly *Neoechinorhynchus manubriensis* Amin, Ha & Ha, 2011), from caroun croaker, *Johnius carouna* (Cuvier), flower croaker, *Nibea albiflora* (Richardson), and silver croaker, *Pennabia argentata* (Houttuyen) (Sciaenidae) in Halong Bay, Vietnam was based on four males and two females (one juvenile and one immature adult). Subsequently, many more specimens became available from *N. albiflora* that were studied using SEM. The new SEM studies revealed many additional features that were not possible to discern with optical microscopy. This new information is reported herein.

## Material and Methods

In July, 2010, many more specimens of *N. manubrianus* became available from new collections from *N. albiflora*, in Halong Bay (107°05’E, 20°45’N). Some of these specimens were stained and examined microscopically, and some were studied by SEM. For SEM studies, 23 specimens from *N. albiflora* that were preserved in 70 % ethanol were placed in critical-point drying baskets and dehydrated using ethanol series of 95 % and 100 % for at least ten minutes per soak followed by critical point drying (Lee, 1992). Samples were mounted on SEM sample mounts, gold coated and observed with a scanning electron microscope (XL30 ESEMFEG; FEI, Hillsboro, Oregon). Digital images of the structures were obtained using digital imaging software attached to a computer.

## Results and discussion

### General observations

**Figs 1–6.**
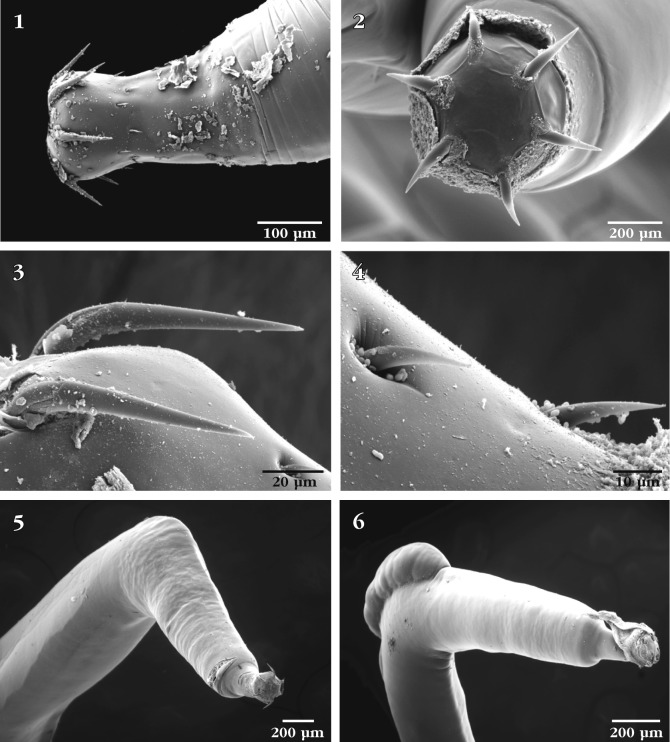
SEM of *Neoechinorhynchus manubrianus*: 1. The proboscis of a female specimen; 2. Apical view of the same proboscis in Fig. 1; 3. Large anterior proboscis hooks; 4. Middle and posterior proboscis hooks; 5. Latero-ventral view of the anterior trunk of a female specimen showing straight anterior end and angle with posterior trunk; 6. Latero-dorsal view of the same specimen in Fig. 5, showing the dorsal fin-like hump.

**Figs 7–12.**
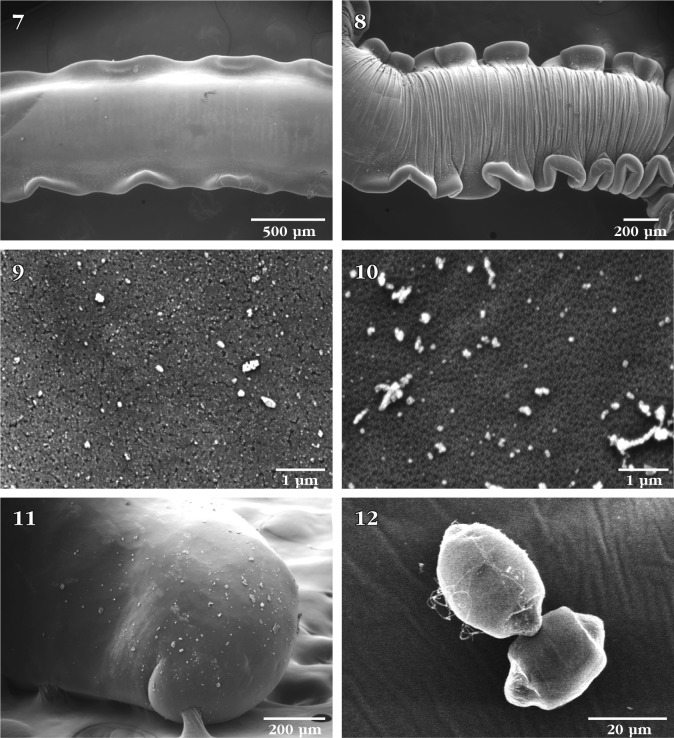
SEM of *Neoechinorhynchus manubrianus*: 7. Mildly undulating trunk fins in a relaxed specimen; 8. A contracted specimen with strongly undulating trunk fins; note contraction lines on the trunk; 9. Epidermal micropores in anterior trunk; 10. Epidermal micropores in posterior trunk; 11. Ventral lateral view of the posterior end of a female specimen; 12. Eggs from a gravid female.

**Figs 13–16.**
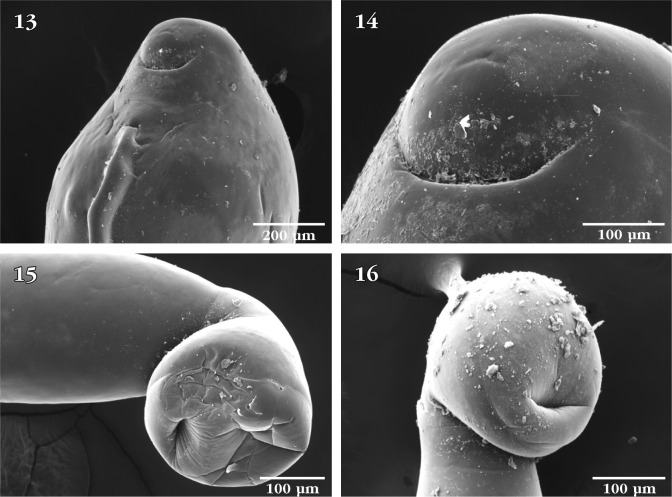
SEM of *Neoechinorhynchus manubrianus*: 13. Ventral view of the posterior end of a female specimen showing the position of the gonopore; 14. Enlargement of the posterior end of specimen in Fig. 13, showing the near terminal subterminal position of the crescent-shaped gonopore; 15. Ventro-lateral view of the bursa of a male specimen showing its bland appearance and articulation at an angle from the posterior trunk; 16. Near-ventral view of the same bursa in Fig. 15, showing its non-central orifice.

Findings of the SEM study (Figs 1–16) were based on adult males and gravid females from which eggs were available. The fin-like protrusions observed in the new material (Figs 6–8) were only observable in whole mounted specimens used in the description (Amin *et al.*, 2011) and those from the more recently acquired specimens as thickenings in the body wall. Eggs (Fig. 12) were not previously described, as no gravid females were available then. Microscopical examination of eggs from the new collection demonstrated the presence of polar prolongation of fertilization membrane, which places *N. manubrianus* in the subgenus *Hebesoma* according to Amin (2002). The morphology of the genital orifices and position of female gonopore were not readily observable in microscopical preparations even though the female opening was described as “terminal” (Amin *et al.*, 2011).

### SEM Observations

The drum-shaped proboscis (Fig. 1) has a flat bare apical end with no protruding apical structures or pores (Fig. 2). Anterior proboscis hooks are long and slender (Fig. 3) and somewhat distant from the considerably smaller and equal middle and posterior hooks (Fig. 4). Anterior trunk straight and invariably sharply angled against the remaining posterior part of the trunk (Fig. 5). An anterior dorsal hump or finlike protrusion is always present at this angled site (Fig. 6). Lateral fin-like protrusions along most of the trunk undulate with the state of relaxation (Fig. 7) and contraction (Fig. 8) of the body. Micropores vary in size and distribution from anterior trunk (Fig. 9) to posterior trunk (Fig. 10). See Amin *et al.* (2009) for implications to differential absorption. The terminal position of the crescent-shaped female gonopore (Fig. 11) is shown from ventral perspective (Figs 13, 14) suggesting a near-terminal position at the bluntly pointed posterior end of the female trunk. The eggs are uniquely shaped (Fig. 12) and are basically cylindrical with two conically shaped polar ends where polar prolongation of fertilization membrane was demonstrated in stained mounts microscopically. The bursa is bland, highly muscular at right angle from the trunk, and not exhibiting a central opening, sensory structures, or any other characteristic features (Figs 15, 16). The uniqueness of *N. manubrianus* rests with the large anterior hook manubria (Amin *et al.*, 2011), anterior angulation of the trunk, trunk fins, and egg shape.
